# An Epidemiologic Study of the Incidence and Mortality of Abdominal Aortic Aneurysms in Koreans Aged ≥50 Years from 2009 to 2018 Based on a National Database

**DOI:** 10.3390/jcm12134319

**Published:** 2023-06-27

**Authors:** Hyangkyoung Kim, Tae-Won Kwon, Yong-Pil Cho, Jun Gyo Gwon, Youngjin Han, Sang Ah Lee, Ye-Jee Kim, Seonok Kim

**Affiliations:** 1 Department of Surgery, College of Medicine, Ewha Womans University, Seoul 07985, Republic of Korea; hkkim77@ewha.ac.kr; 2 Division of Vascular Surgery, Department of Surgery, College of Medicine, University of Ulsan, Asan Medical Center, Seoul 05505, Republic of Korea; ypcho@amc.seoul.kr (Y.-P.C.); doctorgjg@gmail.com (J.G.G.); medjin00@naver.com (Y.H.); ddog94@hanmail.net (S.A.L.); 3 Department of Acute Care Surgery, Korea University Guro Hospital, Seoul 08308, Republic of Korea; 4 Armed Forces Trauma Center, Seongnam 13574, Republic of Korea; 5 Department of Clinical Epidemiology and Biostatistics, College of Medicine, University of Ulsan, Asan Medical Center, Seoul 05505, Republic of Korea; kimyejee@amc.seoul.kr (Y.-J.K.); seonok@amc.seoul.kr (S.K.)

**Keywords:** abdominal aortic aneurysm, incidence, mortality, standardized

## Abstract

Large-scale population studies of the incidence of and mortality from abdominal aortic aneurysm (AAA) are needed to develop healthcare policies and priorities. The aim of this study was to estimate the incidence of AAA and the all-cause mortality from it among Koreans aged ≥50 years from 2009 to 2018 using data from the Korean National Health Insurance System Database. The crude and standardized incidence and all-cause mortality of the disease among patients with unruptured AAA were calculated. A total of 73,933 AAA patients were identified. The overall incidence of AAA in adults ≥50 years during the study period was 37.5 per 100,000 population (49.7 per 100,000 in men and 26.8 per 100,000 in women), with an increase from 32.33 per 100,000 persons in 2009 to 46.85 per 100,000 in 2018. The crude all-cause mortality rate of patients with untreated AAA was 21.26/100 person-years in 2009 and 8.87/100 person-years in 2018, with decreasing trends observed both in men and women. This nationwide study showed that the incidence of AAA in Koreans aged ≥50 years in 2018 was 63.40 per 100,000 in men and 32.07 per 100,000 in women. The overall rates were 0.06% and 0.03%, respectively, with an increasing trend. Mortality has decreased in both treated and untreated patients. The observed increase in incidence suggests a rising burden of AAA in the Korean population, particularly among men. The decreasing mortality rates may indicate improvements in the management and treatment of AAA over the study period.

## 1. Introduction

Abdominal aortic aneurysm (AAA), which is associated with hospitalization, morbidity, and mortality, is a significant public healthcare burden [1]. Studies of the epidemiology of AAA in Western countries show that its incidence and mortality rates increased during the 20th century [2,3]. Because the repair of screen-detected large AAAs reduces peri-operative mortality rates [4], several countries, including Sweden, the UK, the US, and Italy, have introduced cost-effective screening programs for AAA in men aged ≥65 years [5,6,7]. Reports from Sweden, the UK, and the US indicate that both the prevalence of AAA and AAA-specific mortality have declined since 2000, indicating the benefits of these screening programs [8].

To date, few studies have evaluated the incidence of and mortality from AAA in Korea, which currently lacks a screening program. Several studies have reported that the prevalence of AAA is lower in Asian than in Western populations. Large-scale population studies of the incidence of and mortality from AAA are needed, as they can provide insight into the burden of this disease, guide screening and risk prevention priorities and policies, and suggest treatment strategies. The National Health Insurance Service (NHIS) database in South Korea contains complete data on the diagnosis, treatment, and mortality of all residents of the country. Therefore, the aim of this study was to analyze the NHIS data to determine the incidence of AAA and its all-cause mortality from 2009 to 2018.

## 2. Materials and Methods

In this retrospective cross-sectional study, the NHIS database was searched to identify all Korean patients aged ≥50 years who underwent endovascular or open repair of an AAA between 2008 and 2019. Approval for data collection and publication was granted by the institutional review board (IRB No. 2020-1242) of the Asan Medical Centre, which waived the requirement for written informed consent because of the retrospective nature of this study. All procedures were performed in accordance with relevant guidelines and regulations.

The demographic characteristics of each patient aged ≥50 years with AAA were recorded, as were their International Classification of Disease, Tenth Revision (ICD-10), diagnosis codes, procedure codes, prescriptions, and survival as both inpatients and outpatients.

**Figure 1:**
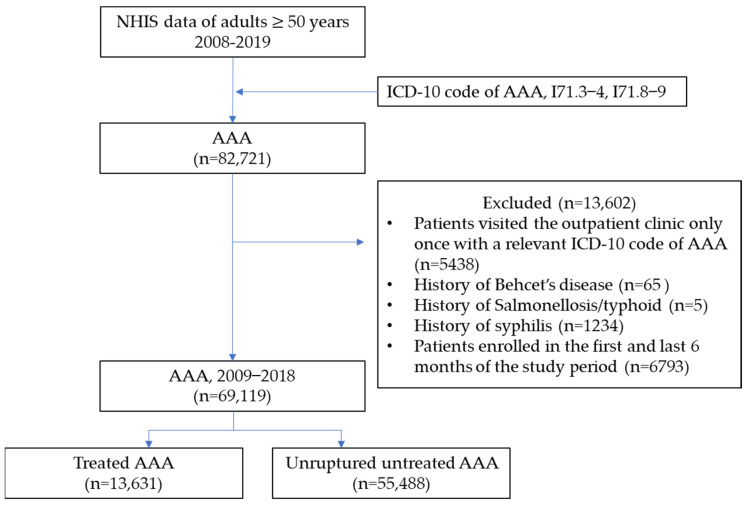
Flow diagram. NHIS, National Health Insurance Service; AAA, abdominal aortic aneurysm; ICD, International Classification of Disease.

The study flow diagram is depicted in Figure 1. Patients aged ≥50 years in the NHIS database who had been diagnosed with AAA (ICD-10 codes I71.3-4 and I71.8-71.9) between January 2008 and December 2019 were initially selected. Patients who had visited the outpatient clinic only once with a relevant ICD-10 code for AAA were excluded. Furthermore, to limit the study to patients with the degenerative type of AAA, those with Behcet’s disease (ICD-10 code M35.2) were excluded. The washout period was defined as the first and last years of the study period. The index date was defined as the first date of AAA diagnosis.

The main study outcomes were the incidence and mortality rates of AAA. The yearly incidence rate of AAA was calculated by dividing the number of patients newly diagnosed with AAA during each calendar year by the total number of patients at risk living in Korea during that calendar year. Exact confidence intervals for incidence were calculated using Ulm’s formula [9]. Both crude and standardized incidence rates were calculated. The standardized incidence rates per 100,000 inhabitants were calculated by applying direct age and sex standardization to the Korean population in 2005 with data obtained from the Korean Statistical Information Service (http://kosis.kr (accessed on 6 September 2021)). All-cause mortality rates, both crude and standardized, were calculated among patients with unruptured AAA. The rates were analyzed by 5-year age groups and different procedure types, including endovascular aneurysm repair (EVAR) and open surgical repair (OSR). Crude mortality rates were calculated by dividing the overall number of deaths in the at-risk AAA population each year (sum of person-years). Standardized mortality ratios (SMRs) were calculated by an indirect method and by determining the ratio of the number of observed deaths to the number of expected deaths, based on the sex- and age-specific general mortality rates during that calendar year in the total Korean population [10]. The total numbers of expected deaths were calculated using Equation (1):

Expected death Total = ∑ (Mortality rate calendar year × Observed duration calendar year/100,000)(1)

All statistical analyses were performed using SAS Enterprise guide version 7.1 software (SAS Institute Inc., Cary, NC, USA).

## 3. Results

### 3.1. Incidence

A search of the NHIS database identified 73,933 patients diagnosed with AAA from 2009 to 2018. The gender-specific annual incidences of AAA are shown in Table 1. The overall incidence of AAA in adults aged ≥50 years during the study period was 37.5 per 100,000 population, 49.7 per 100,000 in men and 26.8 per 100,000 in women. The annual incidence of AAA in Korea increased from 32.33 per 100,000 persons in 2009 to 46.85 per 100,000 populations in 2018. Similarly, the annual incidence of AAA increased both in men and women, from 40.29 per 100,000 in 2008 to 63.40 per 100,000 in 2018 in men and from 25.50 per 100,000 in 2008 to 32.07 per 100,000 in 2018 in women. The incidence increased by approximately 2% to 4% every year from 2008 to 2019.

**Figure 2:**
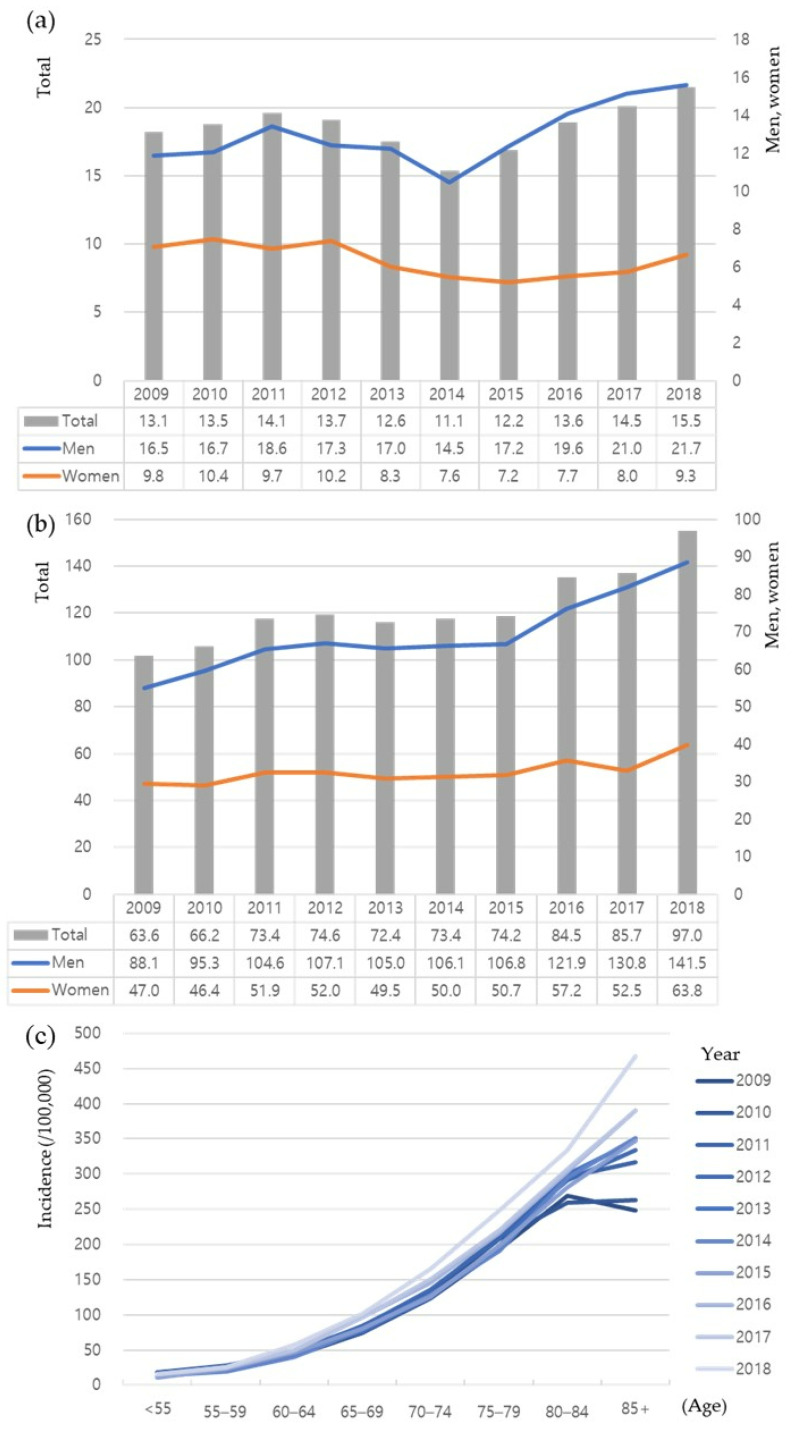
Annual trend of incidence of abdominal aortic aneurysm (AAA) (per 100,000 persons) from 2009 to 2018 in patients aged (**a**) <65 years, and (**b**) ≥65 years. (**c**) Annual age-specific incidence of AAA. The gender-specific annual incidence of AAA tended to increase both in patients aged <65 and ≥65 years, and the age-specific incidence of AAA among patients aged 80 and above has shown an upward trend over time, resulting in a diverging incidence curve. ‘Total’ in panel (**a**,**b**) represents the combined data both for men and women.

**Figure 3:**
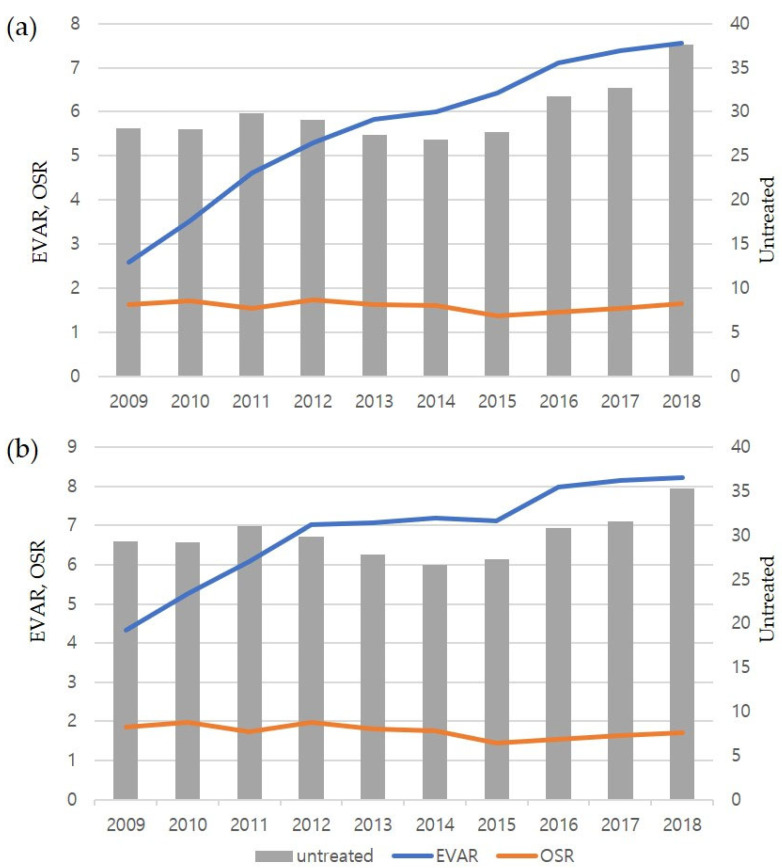
Annual crude (**a**) and standardized (**b**) incidences (per 100,000 persons) from 2009 to 2018 of patients with abdominal aortic aneurysm who underwent endovascular aneurysm repair (EVAR), underwent open surgical repair (OSR), or were untreated. The incidence of EVAR steadily increased during the study period, whereas the incidence of OSR remained constant.

The standardized incidence of AAA also increased both in men and women, from 45.60 per 100,000 in 2008 to 64.46 per 100,000 in 2018 in men and from 22.70 per 100,000 in 2008 to 24.97 per 100,000 in 2018 in women. When compared with the crude incidence, the standardized incidence was slightly lower from 2012 to 2015 than in 2011 in men and from 2012 to 2017 than in 2011 in women. The increases in 2017 and 2018 were most pronounced both in men and women. The gender-specific annual incidence of AAA tended to increase both in patients aged <65 and ≥65 years (Figure 2a,b). In 2018, the incidence of AAA in patients aged ≥65 years was 141.5 per 100,000 in men and 63.8 per 100,000 in women. The increased annual incidence of AAA was more apparent in patients aged ≥65 years than in those aged <65 years and in men than in women. The incidence in women aged <65 years was relatively constant, but slightly increased in women aged ≥65 years. In the later years of our study, the incidence peaked at age 80–84 years, but the incidence of AAA among patients aged 80 and above has shown an upward trend over time, resulting in a diverging incidence curve (Figure 2c). The crude and standardized incidences of untreated AAA were higher than those of treated AAA, with the former showing increasing trends in the three most recent years (Figure 3). The incidence of EVAR steadily increased during the study period, whereas the incidence of OSR remained constant.

**Figure 4:**
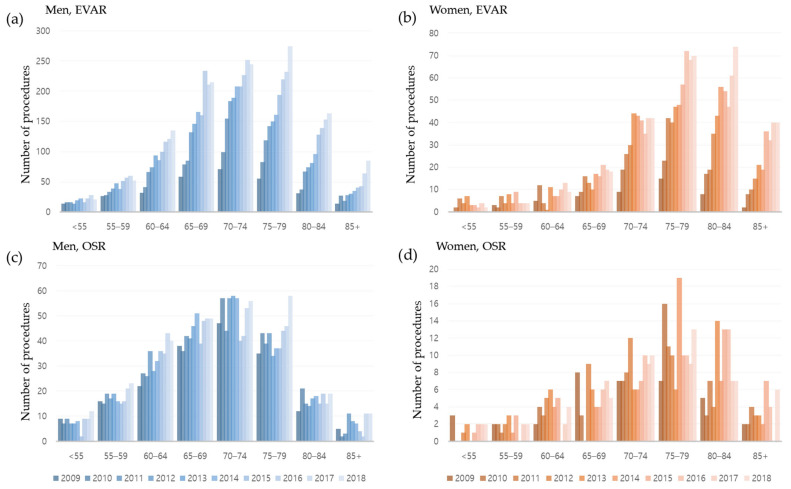
Numbers of (**a**,**c**) men and (**b**,**d**) women who underwent (**a**,**b**) endovascular aneurysm repair (EVAR) and (**c**,**d**) open surgical repair (OSR) of abdominal aortic aneurysm by age group. The increases in EVAR were most apparent among men aged 75–79 years and women aged 80–84 years, whereas the numbers of OSR procedures increased in men in almost all age groups.

The number of procedures in all age groups increased from 2009 to 2018 (Figure 4). The increases in EVAR were most apparent among men aged 75–79 years and women aged 80–84 years (Figure 4a,b), whereas the numbers of OSR procedures increased in men in almost all age groups (Figure 4c). However, the numbers of OSR procedures in most age groups of women peaked during the middle of the study period and then gradually decreased (Figure 4d). Relative to men, the AAA procedure-age curve in women was shifted to the right by approximately 5 years.

### 3.2. Mortality

The crude all-cause mortality rates among patients with unruptured AAA were analyzed separately in treated and untreated patients. The crude all-cause mortality rate of patients with untreated AAA were 21.26/100 person-years in 2009 and 8.87/100 person-years in 2018, with decreasing trends observed both in men and women (Table 2). The crude mortality rate was higher in men than in women. SMR was 5.91 in 2009 and 2.32 in 2018, and it was higher in men than in women. 

**Figure 5:**
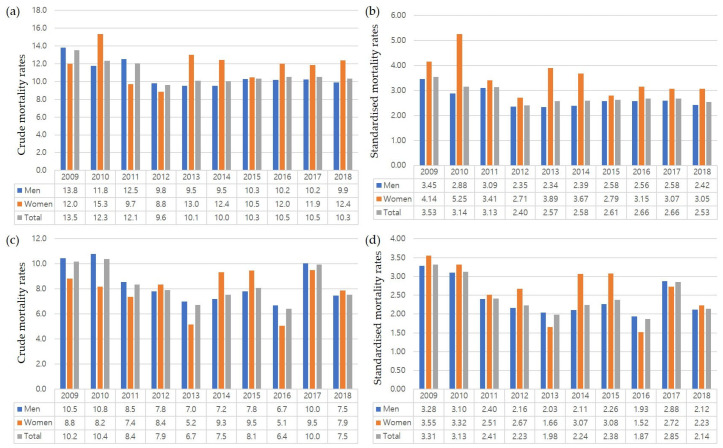
Annual (**a**,**c**) crude and (**b**,**d**) standardized mortality rates (per 100,000 persons) from 2009 to 2018 of patients with abdominal aortic aneurysm who underwent endovascular aneurysm repair (EVAR) (**a**,**b**) and open surgical repair (OSR) (**c**,**d**). The all-cause mortality rate in patients who underwent EVAR decreased from 2009 to 2012, remaining constant thereafter, and the all-cause mortality rate in patients who underwent OSR was lower in 2018 than in 2009.

The crude all-cause mortality rate in patients who underwent EVAR decreased from 2009 to 2012, remaining constant thereafter (Figure 5a). The mortality rate for patients who underwent EVAR was higher among women than men during most of the study period (Figure 5a,b). The crude all-cause mortality rate in patients who underwent OSR was lower in 2018 than in 2009 but varied during the interim (Figure 5c). The difference in mortality rate between men and women who underwent OSR was smaller at the end of the study period (Figure 5c,d).

## 4. Discussion

This nationwide study utilized data from the NHIS to estimate the incidence of AAA and mortality from the disease in Korea from 2009 to 2018. The incidence of AAA in Korea increased during this period for both men and women, with this increasing trend being most apparent in men aged ≥65 years. The incidence of major AAA requiring treatment also increased during the study period. In 2018, the incidence of AAA in subjects aged ≥50 year was 63.40 per 100,000 in men and 32.07 per 100,000 in women, with overall rates of 0.06% and 0.03%, respectively. Higher rates were observed in subjects aged ≥65 years, i.e., 0.14% in men and 0.06% in women. In 2009 and 2010, the incidence of AAA peaked in subjects aged 80–84 ages; in later years, however, the peak incidence was highest in subjects aged ≥85 years, a shift that may be due to the ageing of the Korean population.

Previous studies regarding the epidemiology of AAA in Western countries vary in the definition of AAA and/or the study population. The prevalence of AAA in these studies ranged from 1.3% to 8% [11,12,13,14,15,16], and the annual incidence of newly diagnosed AAA in screened populations ranged from 0.4% to 0.67% [17,18]. Although most Asian countries have not yet adopted a screening program for AAA, making it difficult to determine its exact prevalence, the incidence of AAA is generally thought to be lower in Asian than in Western populations [19]. The incidence of AAA in the present study was much lower than previously reported, a finding likely due to differences in study populations. Because a screening program is not yet available in Korea, the incidence of AAA in the present study was calculated based on diagnosed patients in the NHIS database. The actual incidence of AAA in the general population is likely higher. However, even after accounting for missing patients due to the absence of screening, the incidence of AAA was much lower in the present study than in studies from Western countries, suggesting that this difference may be due, at least in part, to racial/ethnic differences. Accurate determination of the incidence of AAA in the general population, based on a screening program, may be required to evaluate the socioeconomic burden of AAA in Korea. As observed in Figure 2c, a notable phenomenon emerges: the incidence of AAA among patients aged 80 and above has shown an upward trend over time, contrasting with its lower occurrence in the past. This can be attributed to the significant aging population in Korea, specifically with the increasing life expectancy. Consequently, it can be interpreted that the incidence of AAA is on the rise within the elderly demographic.

Despite the increased incidence, mortality rates decreased over time in both treated and untreated AAA patients in this study. Mortality from AAA in developed countries has decreased since the late 1990s [20,21]. AAA is associated with multiple risk factors, with tobacco smoking being the main modifiable risk factor for its development and rupture [22]. The decrease in AAA correlated with reductions in cardiovascular risk factors, due in part to smoking cessation and treatment with cardiovascular drugs [21,23]. The mortality rate associated with the elective treatment of non-ruptured AAA has decreased in recent years [24], due in part to improvements in the treatment or adoption of endovascular techniques [25]. Similarly, the present study found that outcomes improved with the treatment of non-ruptured AAA. Moreover, the study found that reductions in mortality rates were greater in untreated AAA patients than in treated ones. This suggests that managing related diseases or risk factors for AAA may have a greater impact on mortality improvement than treating the AAA itself. As stated in the guidelines, identification of these factors will play a major role in improving the prognosis of patients with AAA [26].

The major strength of this study was its complete coverage of the Korean population since incidence and mortality trends in patients with unruptured AAA were based on data from a compulsory national insurance system. Nevertheless, this study had several important limitations. Patients with ruptured AAA were not included because accurate statistics could not be obtained on out-of-hospital deaths or pre-hospital deaths with obscure diagnoses. The incidence of AAA in the general population could not be determined because of the absence of a screening program. Nevertheless, this study provided robust evidence of the incidence of and mortality from AAA in a large Asian population. Knowledge of these trends may help guide public health policies and implementation, including the introduction of AAA screening programs and increased focus on risk factor management and early intervention strategies.

## 5. Conclusions

In 2018, the incidence of AAA in Koreans aged ≥50 years was 0.06% in men and 0.03% in women, with increasing trends. Mortality rates have decreased over time both in treated and untreated AAA. The observed increase in incidence suggests a rising burden of AAA in the Korean population, particularly among men. The decreasing mortality rates may indicate improvements in the management and treatment of AAA over the study period. Further research and healthcare initiatives are warranted to address this public health concern and formulate appropriate strategies for the prevention, diagnosis, and treatment of AAA in Korea.

## Figures and Tables

**Table 1:** Annual and standardized incidence rates (per 100,000 persons) of abdominal aortic aneurysm from 2009 to 2018.

	No. of Patients	Population	Incidence (95% CI)	Standardised Incidence (95% CI)
Total				
2009	4390	13,580,019	32.33 (31.38, 33.30)	68.30 (66.78, 69.83)
2010	4748	14,263,364	33.29 (32.25, 34.25)	70.59 (69.05, 72.16)
2011	5387	14,966,440	35.99 (35.04, 36.97)	76.07 (74.47, 77.69)
2012	5655	15,637,721	36.16 (35.23, 37.12)	75.72 (74.12, 77.34)
2013	5658	16,240,030	34.84 (33.94, 35.76)	72.20 (70.64, 73.78)
2014	5786	16,818,677	34.40 (33.52, 35.3)	69.98 (68.44, 71.53)
2015	6185	17,416,450	35.51 (34.63, 36.41)	71.18 (69.63, 72.75)
2016	7257	17,994,515	40.33 (39.41, 41.27)	79.94 (78.29, 81.60)
2017	7725	18,572,007	41.59 (40.67, 42.53)	81.74 (80.08, 83.42)
2018	8993	19,193,790	46.85 (45.89, 47.83)	89.43 (87.69, 91.19)
Men				
2009	2526	6,269,886	40.29 (38.73, 41.89)	45.60 (44.36, 46.86)
2010	2808	6,605,542	42.51 (40.95, 44.11)	48.01 (46.74, 49.31)
2011	3228	6,951,176	46.44 (44.85, 48.07)	52.40 (51.07, 53.75)
2012	3381	7,285,950	46.40 (44.85, 48.00)	52.06 (50.73, 53.40)
2013	3482	7,590,057	45.88 (44.36, 47.43)	51.04 (49.73, 52.38)
2014	3548	7,880,430	45.02 (43.55, 46.53)	49.47 (48.18, 50.78)
2015	3865	8,176,100	47.27 (45.79, 48.79)	50.77 (49.46, 52.10)
2016	4583	8,467,751	54.12 (52.57, 55.71)	57.29 (55.90, 58.70)
2017	5130	8,756,242	58.59 (56.99, 60.21)	60.73 (59.30, 62.18)
2018	5743	9,059,019	63.40 (61.77, 65.06)	64.46 (62.98, 65.95)
Women				
2009	1864	7,310,133	25.50 (24.35, 26.68)	22.70 (21.83, 23.59)
2010	1940	7,657,823	25.33 (24.22, 26.49)	22.58 (21.71, 23.47)
2011	2159	8,015,264	26.94 (25.81, 28.10)	23.67 (22.78, 24.58)
2012	2274	8,351,771	27.23 (26.12, 28.37)	23.66 (22.77, 24.57)
2013	2176	8,649,974	25.16 (24.11, 26.24)	21.16 (20.32, 22.02)
2014	2238	8,938,248	25.04 (24.01, 26.10)	20.51 (19.68, 21.36)
2015	2320	9,240,351	25.11 (24.10, 26.15)	20.42 (19.59, 21.26)
2016	2674	9,526,765	28.07 (27.01, 29.15)	22.64 (21.77, 23.54)
2017	2595	9,815,766	26.44 (25.43, 27.47)	21.01 (20.17, 21.87)
2018	3250	10,134,772	32.07 (30.97, 33.19)	24.97 (24.06, 25.91)

**Table 2:** Annual crude and standardized mortality rates of patients with untreated unruptured abdominal aortic aneurysm from 2009 to 2018.

	Observed Deaths	Person-Year	Crude Mortality per 100 Person-Years	Standardised Mortality Ratio (95% CI)
Total				
2009	611	2874.4	21.26	5.91 (5.45–6.40)
2010	906	5946.9	15.23	4.28 (4.01–4.57)
2011	1189	8968.3	13.26	3.77 (3.56–3.99)
2012	1391	12,280.2	11.33	3.13 (2.97–3.30)
2013	1562	15,079.2	10.36	2.92 (2.77–3.07)
2014	1722	17,786.8	9.68	2.73 (2.60–2.86)
2015	2007	20,450.1	9.81	2.68 (2.57–2.80)
2016	2237	23,554.7	9.50	2.58 (2.47–2.69)
2017	2367	26,903.5	8.80	2.36 (2.26–2.45)
2018	2729	30,779.1	8.87	2.32 (2.23–2.40)
Men				
2009	332	1529.6	21.71	5.34 (4.78–5.94)
2010	538	3150.7	17.08	4.24 (3.89–4.61)
2011	725	4758.3	15.24	3.84 (3.57–4.13)
2012	821	6469.1	12.69	3.14 (2.93–3.37)
2013	902	7983.7	11.30	2.86 (2.68–3.06)
2014	982	9462.5	10.38	2.66 (2.50–2.83)
2015	1110	10,977.3	10.11	2.56 (2.41–2.72)
2016	1297	12,813.0	10.12	2.57 (2.43–2.72)
2017	1378	14,802.6	9.31	2.38 (2.26–2.51)
2018	1610	17,220.4	9.35	2.35 (2.23–2.46)
Women				
2009	279	1344.8	20.75	6.79 (6.02–7.63)
2010	368	2796.2	13.16	4.34 (3.91–4.81)
2011	464	4210.0	11.02	3.65 (3.33–4.00)
2012	570	5811.1	9.81	3.11 (2.86–3.38)
2013	660	7095.6	9.30	2.99 (2.77–3.23)
2014	740	8324.4	8.89	2.83 (2.63–3.04)
2015	897	9472.8	9.47	2.85 (2.67–3.05)
2016	940	10,741.7	8.75	2.59 (2.43–2.76)
2017	989	12,100.9	8.17	2.32 (2.18–2.47)
2018	1119	13,558.7	8.25	2.27 (2.14–2.41)

## Data Availability

The data that support the findings of this study are available from National Health Insurance Service of Korea, but restrictions apply to the availability of these data, which were used under license for the current study and so are not publicly available.
